# Abnormal number cell division of human thyroid anaplastic carcinoma cell line, SW 1736

**DOI:** 10.1016/j.dib.2015.09.030

**Published:** 2015-10-09

**Authors:** Keiichi Ikeda, Toshiaki Tachibana, Yuta Suzuki, Kouki Fujioka, Hiroshi Takeyama, Yoshinobu Manome

**Affiliations:** a Division of Molecular Cell Biology, Core Research Facilities for Basic Science, Research Center for Medical Sciences, The Jikei University School of Medicine, Tokyo, Japan; b Department of Surgery, The Jikei University School of Medicine, Tokyo, Japan

**Keywords:** Thyroid cancer, Cell division, Live cell imaging

## Abstract

Cell division, during which a mother cell usually divides into two daughter cells during one cell cycle, is the most important physiological event of cell biology. We observed one-to-four cell division during imaging of live SW1736 human thyroid anaplastic carcinoma cells transfected with a plasmid expressing the hybrid protein of green fluorescent protein and histone 2B (plasmid eGFP-H2B). Analysis of the images revealed a mother cell divided into four daughter cells. And one of the abnormally divided daughter cells subsequently formed a dinucleate cell.

**Specifications Table**

**Table:** 

Subject area	*Biology*
More specific subject area	*Cell culture*
Type of data	*Image (microscopy)*
How data was acquired	*Microscope*
Data format	*Processed image*
Experimental factors	*Transfection with eGFP-H2B plasmid*
Experimental features	*Abnormal cell division was accidentally and clearly recorded.*
Data source location	*Tokyo, Japan*
Data accessibility	*Data are provided in this article*

**Value of the data**

• During live cell imaging, we encountered a one-to-four cell division of the human thyroid anaplastic carcinoma cell line, SW1736.
• All daughter cells of the cell division were viable.
• One of the daughter cells formed a dinucleated cell.

## 1 Data, experimental design, materials and methods

**Fig. 1:**
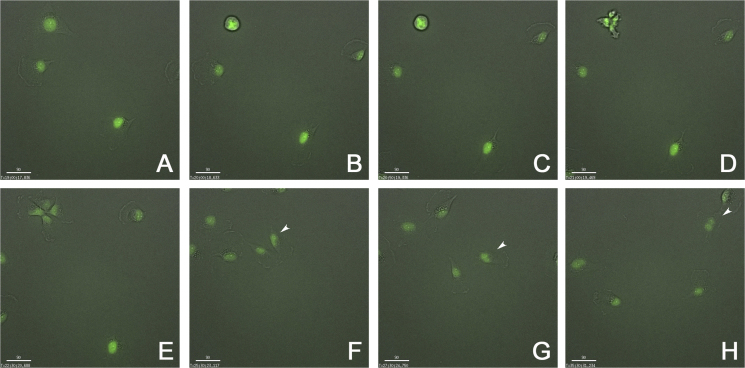
Time-laps images of SW1736 human thyroid anaplastic carcinoma cells (objective lens: ×20). Image at 19 h (A), 20 h (B), 20 h and 50 min, just before cell division (C), 21 h, just after cell division was started (D), 22 h and 30 min (E), 25 h and 30 min (F), 27 h and 30 min (G), and 35 h and 30 min (H) after acquisition of images had started. Arrowheads in F–H indicate a dinuleated daughter cell. A size bar indicates 90 μm.

**Fig. 2:**
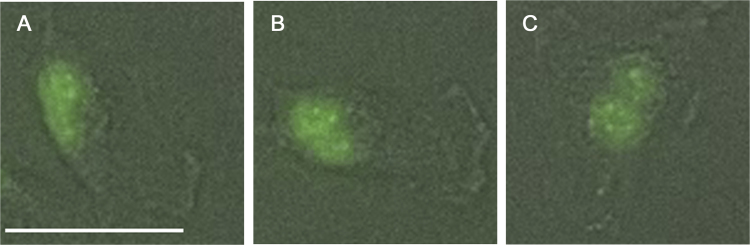
Digitally-zoomed in image processed by Adobe Photoshop CS5 Extended version 12.0.4x64 software (Adobe Systems, Inc., San Jose, CA, USA) of a daughter cell which was indicated by an arrow head in Fig. 1 and formed a dinucleated cell. A daughter cell at 25 h and 30 min (A), 27 h and 30 min (B), and 35 h and 30 min (C) after acquisition of images had started. A size bar indicates 90 μm.

During live cell imaging, SW 1736 human thyroid anaplastic carcinoma cells transfected with the eGFP-H2B plasmid, was divided from one to four cells as shown in Fig. 1 and supplementary video. All daughter cells were alive during imaging and one of the daughter cells formed a dinucleated cell (Fig. 1F–H and Fig. 2).

Supplementary material related to this article can be found online at 10.1016/j.dib.2015.09.030

The following is the Supplementary material related to this article Video 1.

Video 1 The video 1 of time-laps imaging of SW1736 human thyroid anaplastic carcinoma cells transfected with eGFP-H2B plasmid. Images were acquired by differential interference contrast and green fluorescent conditions every 10 min for 35 h and 30 min. A size bar indicates 90 μm.

### 1.1 Cell culture, transfection of the eGFP-H2B plasmid

SW 1736 human thyroid anaplastic carcinoma cells (provided by the Memorial Sloan-Kettering Cancer Center, New York, NY, USA), were cultivated as previously described [1], [2], [3]. The eGFP-H2B plasmid was constructed by cloning of the human H2B DNA into plasmid pEGFP-C1 (Clontech Laboratories, Inc., Mounain View, CA, USA). The eGFP-H2B plasmid was transfected i\nto SW1736 human thyroid anaplastic carcinoma cells by electroporation with Gene Pulser II Electroporation System (Bio-Rad Laboratories, Inc., Hercules, CA, USA) and the transfected cells were selected with G418 disulfate salt (Sigma-Aldrich Co., St. Louis, MO, USA).

### 1.2 Imaging of transfected SW1736 SW 1736 human thyroid anaplastic carcinoma cells

Live cell imaging of transfected SW1736 human thyroid anaplastic carcinoma cells was performed as follows. A 35 mm glass base culture dish containing cells to be imaged was then placed onto the stage of a DeltaVision Core-SP microscope (Berthold Australia Pty Ltd. [Applied Precision], Bundoora, Victoria, Australia) and kept at 37 °C in a humidified 95% (v/v) O_2_ containing 5% (v/v) CO_2_ gas mixture during imaging. Time-laps Images of the cells were acquired by differential interference contrast and green fluorescent images using a fluorescein isothiocyanate filter set (ex: 490 [20] nm/em: 525 [36] nm) every 10 min. The images obtained were processed and video data were exported by softWoRx version 4.0.0. Release 16 (Berthold Australia Pty Ltd. [Applied Precision]).
